# Core Mycorrhizal Fungi Promote Seedling Growth in *Dendrobium officinale*: An Important Medicinal Orchid

**DOI:** 10.3390/plants14071024

**Published:** 2025-03-25

**Authors:** Yi-Hua Wu, Xiang-Gui Chen, Neng-Qi Li, Tai-Qiang Li, Rengasamy Anbazhakan, Jiang-Yun Gao

**Affiliations:** State Key Laboratory for Vegetation Structure, Functions and Construction, Ministry of Education Key Laboratory for Transboundary Ecosecurity of Southwest China, Institute of Biodiversity, School of Ecology and Environmental Science, Yunnan University, Kunming 650500, China

**Keywords:** seedling-associated mycorrhizal fungi, growth-promoting fungi, seedling growth, synthetic fungal combinations, synergistic/offset effects

## Abstract

The critically endangered orchid *Dendrobium officinale*, valued for its medicinal properties, depends on specific seedling-associated mycorrhizal fungi (SAMF) for successful early-stage seedling development. However, conservation efforts are often hindered by difficulties in obtaining suitable SAMF, leading to poor seedling establishment in both natural and cultivated environments. In this study, we explored the growth-promoting effects of SAMF and evaluated the performances of synthetic fungal combinations. Our results demonstrated that mycorrhizal fungi, widely distributed across multiple habitats with high isolation frequencies, significantly promoted the growth of *D. officinale*, with specific fungi favoring different growth parameters. *Tulasnella* sp. TP-2 and TP-3 significantly improved stem diameter and plant height by 2.622 mm and 4.621 cm, while *Tulasnella* sp. TP-8 significantly increased tillering by a factor of 4.47. Additionally, *Tulasnella* sp. TP-11 and TP-13 markedly increased the number of new leaves (4.45) and new roots (2.688), respectively, identifying them as essential core OMFs for *D. officinale* seedlings. Contrary to expectations, synthetic fungal combinations composed of core orchid mycorrhizal fungi (core OMFs) did not exhibit synergistic growth-promoting effects. Instead, pronounced offset effects were observed, indicating that interactions between fungi may introduce competition or inhibition, limiting their collective ability to enhance plant growth. Our results confirmed that the core OMFs significantly promoted the growth of *D. officinale* seedlings. These core OMFs can serve as essential components in specialized microbial fertilizers for *D. officinale*, improving growth efficiency and yield, and supporting the sustainable development of the *D. officinale* industry.

## 1. Introduction

Mycorrhizal symbiosis plays a pivotal role in the survival and ecological adaptability of orchids [1]. By forming partnerships with mycorrhizal fungi, orchids can access vital nutrients from the environment, increase resistance to pathogens, and enhance their resilience to environmental stress [2,3,4,5]. This symbiotic relationship is dynamic, with orchids adjusting their fungal community composition in response to different growth and developmental stages, such as growth from germination to adulthood [3,6]. For instance, *Gastrodia elata* seeds depend on *Mycena* fungi for germination but require *Armillaria mellea* for subsequent growth [7]. Similarly, *Anacamptis papilionacea* shifts from *Tulasnella calospora*-driven germination to *Ceratobasidium*-supported seedling development [8]. It suggests that switching fungal partners in response to changing physiological needs provides significant ecological adaptability [3,9,10].

Previous studies on *Dendrobium officinale* have identified key fungal partners (e.g., *Epulorhiza*, *Tulasnella*, and Sebacinales) that enhance seed germination [11,12], nitrogen [13] uptake, and polysaccharide accumulation [14]. For instance, Zhang et al. demonstrated that *Tulasnella calospora* significantly promoted root and leaf growth in *D. officinale*, increasing biomass (155.2%) and photosynthetic pigment content by 99.6% compared to controls. Total medicinal polysaccharide content also increased by 42.69% under fungal symbiosis [14]. Additionally, Sebacinales LQ strains isolated from both adult roots and protocorms markedly improved seed germination rates, reaching 84% after 30 days of symbiosis [11]. In our *D. officinale* reintroduction practices using the fungus–seed bag method, we observed that the seeds rapidly germinated and formed protocorms by germination-promoting fungi. But, due to the lack of suitable SAMF, subsequent seedling development was often stunted or led to mortality. However, most studies have focused on mycorrhizal fungi diversity in adult orchids and during seed germination, leaving a critical knowledge gap regarding the seedling-associated mycorrhizal fungi (SAMF). *In situ/ex situ* baiting seedling mycorrhizae of *D. officinale* revealed that SAMF differ significantly from those in adult plants and germinating seeds [15]. The functional divergence of mycorrhizal communities across the developmental stages of *D. officinale* may reflect adaptive strategies to distinct nutritional demands and habitat constraints [16]. Germinating seeds and seedlings prioritize carbon acquisition via Serendipitaceae-dominated symbionts, while adult plants favor Tulasnellaceae for sustained nutrient uptake in epiphytic niches [16,17]. Host-selective signaling and fungal niche partitioning further reinforce this dynamic [4]. This highlights the specific roles and adaptations of mycorrhizal fungi at different developmental stages of orchids [18,19].

Environmental factors, such as soil composition, seasonal variations, and the presence of competing plant species, significantly affect the composition of the fungal communities associated with orchids [3,20]. These factors not only shape the diversity of fungal partners but can also alter the cost–benefit dynamics of symbiosis, requiring habitat-specific adaptations in conservation approaches [3,21]. For example, *Goodyera pubescens* can adapt to extremely dry environments by forming new associations with different fungal partners [10]. Remarkably, despite habitat differences in fungal community composition, core fungal taxa were prevalent across habitats. This phenomenon was exemplified by *Cyrtochilum retusum* and *Epidendrum macrum*, which maintained a core of keystone orchid mycorrhizal fungi (OMFs) that were ubiquitously distributed stably across temporal change, and a majority of OMFs were randomly associated [22]. Similar phenomena were found in our previous study; we collected substrates from six *D. officinale* habitats to bait seedling mycorrhizas and successfully isolated and obtained 27 SAMF. These fungi spanned multiple genera, including *Tulasnella*, *Thanatephorus*, *Trichoderma*, *Pletosphaerella*, *Fusarium*, and members of Serendipitaceae. Most fungal strains exhibited limited habitat specificity, being isolated from no more than three habitats. For instance, three *Tulasnella* sp. (TP-4, TP-5, and TP-6) and two species of Serendipitaceae (TP-14 and TP-16) were isolated from two habitats. *Thanatephorus* sp. TP-17 and *Trichoderma* sp. TP-24 were found in three habitats. In contrast, five specific mycorrhizal fungi (*Tulasnella* sp. TP-2, TP-8, TP-9, TP-11, and TP-13) were consistently present and frequently isolated, suggesting that they serve as the dominant fungi critical to orchid growth across various habitats [23]. These dominant fungi likely perform irreplaceable functions, acting as the core mycorrhizal fungi that support orchid survival and adaptability. Understanding their ecological roles is essential for devising evidence-based strategies for orchid conservation and sustainable utilization.

In natural ecosystems, plants interact with various microbial taxa, leading to either positive or negative interactions [24,25,26]. Previous studies have predominantly focused on the interaction between single fungal species and orchids, confirming that certain fungal strains can promote orchid growth at specific stages [8,11,15,27]. However, the ecological functions of natural communities often rely on the synergistic interactions of multiple mycorrhizal fungi—different fungi form functional enhancement networks via niche differentiation and nutrient complementarity [28,29,30]. This suggests that synthetic fungal communities may more efficiently exploit limited resources and enhance plant growth [8]. Empirical evidence from *D. officinale* confirms high mycorrhizal fungi diversity, with multiple fungi promoting both germination and growth [11,27,31]. Nevertheless, key scientific questions remain unresolved. Do synthetic fungal combinations composed of growth-promoting strains yield emergent synergies? Does functional redundancy among co-occurring fungi intensify resource competition? The resolution of these questions remains pivotal for optimizing mycorrhizal resource utilization in conservation biotechnologies.

This study focuses on SAMF in *D. officinale*, addressing the following questions. (a) How do fungal habitat origin and isolation frequency influence seedling performance? (b) What are the functional differences among the various SAMFs in promoting seedling growth? (c) Do synthetic fungal combinations enhance seedling growth more than single fungal species? The study aims to provide effective fungal resources for the artificial cultivation of *D. officinale* while establishing a theoretical foundation for applying microbiome engineering to endangered plant species conservation.

## 2. Materials and Methods

### 2.1. Study Species

*D. officinale*, a widely used orchid in traditional Chinese medicine, is primarily distributed in the subtropical and tropical regions of Southern China, including the provinces of Yunnan, Guangxi, Guizhou, Sichuan, and Zhejiang [23]. Wild *D. officinale* is known for its strict growth requirements, typically thriving on tree trunks or rocks in high-altitude, humid forests. Flowering occurs from March to June, with a fruit set rate ranging from 10% to 17% [32]. In recent years, the sharp increase in market demand has led to severe overharvesting of wild *D. officinale*, causing a significant decline in natural populations and habitat degradation, pushing the species toward endangerment [32]. The Chinese Ministry of Agriculture and Rural Affairs and the National Forestry and Grassland Administration officially released a new version of “The List of National Key Protected Wild Plants” in 2021 (http://www.gov.cn/zhengce/zhengceku/2021-09/09/content_5636409.htm, accessed on 2 January 2025). *D. officinale* is listed as a class II national protected wild plant.

### 2.2. Fungal Strains and Plant Materials

Our research group previously established an optimized protocol for isolating and culturing fungi from the root of *D. officinale* seedlings [23]. The 27 SAMFs used in this study were obtained through *ex situ* seedling baiting with mycorrhizal roots of *D. officinale,* followed by isolation and culture from the colonized roots. The complete protocol was executed as follows. Substrates (mosses, bark, and litters) were collected from six *D. officinale* native habitats, namely Guangnan (Yunnan, GN), Shimian and Luding (Sichuan, SM, and LD), Langshan Mountain (Hunan, KSLM and DXLM), and Luotian (Chongqing, LT). *In vitro*-produced seedlings were transplanted into these substrates for baiting mycorrhizal colonization, followed by fungal isolation from the colonized roots. The 27 SAMFs included 16 OMFs (Tulasnellaceae and Serendipitaceae; TP-1 to TP-16) and 11 non-OMFs (e.g., *Thanatephorus* sp., *Trichoderma* sp., and *Muscodor* sp., TP-17 to TP-27), with the species identities, habitat origins, and isolation frequencies detailed in Table S1 [23]. All fungi were stored in the fungus bank of the Orchid Conservation Group, School of Ecology and Environmental Science, Yunnan University, China. Before symbiotic culture, fungal strains were inoculated on potato dextrose agar medium (PDA; 200 g/L potato, 20 g/L glucose, 20 g/L agar; autoclaved at 121 °C for 20 min; BOXUN YXQ-50ALL, Shanghai, China) according to the procedure in darkness at 25 ± 1 °C for 7 days. The actively growing mycelia from the colony margin were used as the fungal inoculum in subsequent symbiotic cultures.

Mature *D. officinale* capsules were obtained via artificial pollination at a cultivation base in Yueqing, Zhejiang Province, China. The seeds were surface-sterilized and germinated aseptically on an MS medium (BS1025, BAISI, Hangzhou, China; 40 g/L potato extract, 20 g/L sucrose, 2.5 g/L Hyponex No.1, 2 g/L peptone, 2 mL/L 6-BA [1 mg/mL], 0.5 mL/L NAA [1 mg/mL], 6.5 g/L agar). After six months of cultivation, uniform *in vitro*-produced seedlings (4–5 leaves, 2 roots, 1.7–2.2 cm height, and 1.2–1.5 mm stem diameter) were selected for the symbiotic experiments.

### 2.3. Symbiotic Cultures

Based on previous research, oatmeal agar (OMA; 4 g/L oatmeal, 8 g/L agar) was employed as the symbiotic medium. The OMA medium was dispensed into tissue culture bottles (110 ∗ 78 mm; 120 mL per bottle) and autoclaved (121 °C, 103 kPa, 20 min). Five seedlings were transferred into each bottle. Any contaminated bottles were discarded after a one-week adaptation period in the tissue culture room. Fungal inoculation was based on previous methods [33], including a sterile control (CK). Briefly, 1 mL pipette tips were used to excise uniform fungal plugs, which were then centrally positioned equidistant from all seedlings within each bottle. Each treatment was replicated at least 35 times to account for potential contamination (10% contamination rate based on prior trials) and destructive sampling. Three replicates per treatment were randomly selected every 30 days for fungal colonization, resulting in a total loss of 9 replicates over 90 days. This ensured ≥30 valid seedlings for the final statistical analysis. All bottles were maintained in a culture room under controlled environmental conditions: 25 ± 2 °C, a 12/12 h light/dark cycle, and a light intensity of 2000 lux.

After 30 and 90 days, the seedlings were harvested to assess the effects of fungal inoculation. The parameters measured included seedling dry weight (biomass), leaf and root counts, sprouting tillers, plant height, and stem diameter. Dry weight was recorded after oven-drying at 80 °C for 72 h. New leaf and root growth was determined by subtracting the initial counts from the totals at analysis. Sprouting tillers were counted as bud sprouts. Plant height was measured as the stem length, and the stem diameter was taken 1 cm above the stem base. Thirty seedlings per treatment were randomly selected for these assessments. To confirm the mycorrhizal symbiosis, the root segments were sampled at 30, 60, and 90 days post-inoculation to check for fungal colonization. Collectively, these parameters provided a comprehensive assessment of the impact of fungal inoculation on seedling development.

### 2.4. Testing the Ability of Synthetic Fungal Combinations to Promote Seedling Growth

Core fungi (*Tulasnella* sp. TP-2, TP-3, TP-8, TP-11, and TP-13) were selected for synthetic fungal combinations based on their significant enhancement of *D. officinale* seedling growth parameters (e.g., new leaves, new roots, stem diameter, plant height, and tillers) and isolation frequency. It was hypothesized that these fungi act synergistically. To test this, 11 synthetic fungal combinations were designed (Table S2) to improve seedling growth across various parameters. The methodology followed previous growth promotion assessments. The growth parameters were measured from 30 randomly selected seedlings per treatment at 30 and 90 days post-inoculation. Additionally, five root samples per group were ink-stained to check for fungal colonization.

### 2.5. Synergistic and Offset Effects

In order to assess the synergistic and offset effects of each synthetic fungal combination in a quantitative manner, this study utilized a method similar to that outlined by Hori et al. with some minor adjustments [26]. The growth responses of *D. officinale* to the fungal treatments were normalized by calculating a standardized growth index:

(1) $$SG_{T}\left(i\right)=\frac{X_{T}\left(i\right)-\bar{X_{Ck}}}{SDck},$$

where *X_T_(i)* represents the trait measurement for the *i* plant under single or combined fungal inoculations, and $\bar{X_{Ck}}$ and SDcκ denote the control (CK) mean and standard deviation, respectively. For the *D. officinale* seedlings inoculated with synthetic fungal combinations composed of strains *A*, *B*, *C*, *D*, and *E*, the deviation from the maximum effects of single inoculations is calculated as follows:

(2) $${DMX}_{ABCDE}(i)={SG}_{ABCDE}\;(i)-\max(\bar{SG_{A}},\;\bar{SG_{B}},\;\bar{SG_{C}},\;\bar{SG_{D}},\;\bar{SG_{E}}),$$

The value SG*_ABCDE_(i)* signifies the standardized growth index for samples treated with the synthetic fungal combinations, while $\bar{SG_{A}}$, $\bar{SG_{B}}$, $\bar{SG_{C}}$, $\bar{SG_{D}}$, and $\bar{SG_{E}}$ reflect the corresponding indices following single inoculations with fungal strains *A* to *E*. A mean deviation index ($\bar{DMX_{ABCDE}}$) exceeding zero indicates a synergistic effect. Similarly, to evaluate the offset effects of synthetic fungal combinations, the deviation from the minimum effects of single inoculations is defined as follows:

(3) $${DMN}_{ABCDE}(i)=\min(\bar{SG_{A}},\;\bar{SG_{B}},\;\bar{SG_{C}},\;\bar{SG_{D}},\;\bar{SG_{E}})-SG_{ABCDE}(i),$$

A mean offset effect index ($\bar{DMN_{ABCDE}}$) larger than zero signifies an offset in the synthetic fungal combinations.

### 2.6. Detection of Fungal Colonization

After autoclaving (121 °C, 103 kPa, 20 min) in 10% KOH and neutralizing in 2% HCl, the roots were stained with a 10% (*v*/*v*) ink dye solution (comprising 10% Pelikan 4001 Bright Black and 3% acetic acid) and heated at 95 °C for 30 min. The samples were then preserved in 100% lactic acid (PHR1215, Sigma-Aldrich, Shanghai, China) at 4 °C before microscopic examination [34]. Additionally, the roots were fixed in FAA (BL592A, Biosharp, Beijing, China), dehydrated in an ethanol series, infiltrated with ethanol, and embedded in LR White resin (L9774, Sigma-Aldrich, St. Louis, MO, USA). Semi-thin sections (3 μm) were stained with 1% (*w*/*v*) toluidine blue (89640, Sigma-Aldrich, Shanghai) and examined under a light microscope (DM2000, Leica Microsystems GmbH, Wetzlar, Germany) [30].

### 2.7. Statistical Analysis

The study analyzed the significance of the differences in growth parameters among various fungal inoculation treatments and different synthetic fungal combinations inoculation treatments. A one-way ANOVA, followed by Duncan’s multiple range test, was used for normally distributed data, while a generalized linear model (GLM) was employed for non-normally distributed data. The alpha-type I error was set at 5%, with non-significant differences having *p* > 0.05. All statistical analyses and data visualizations were performed using R software (version 4.0.0).

## 3. Results

### 3.1. The Capacity of Fungi to Support Seedling Growth

**Figure 1:**
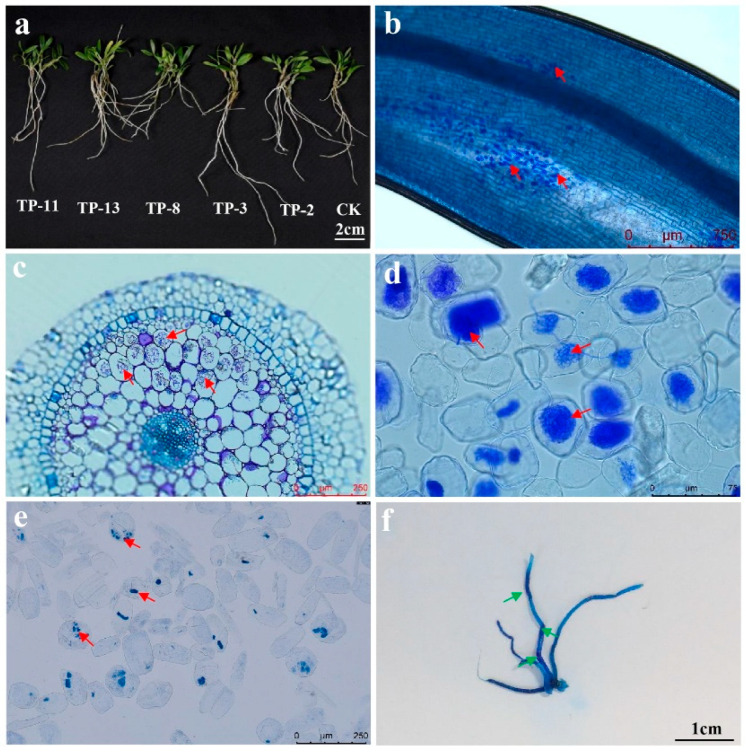
Colonization of mycorrhizal fungi in the roots of *D. officinale* seedlings after symbiotic cultivation. (**a**) Seedlings inoculated with TP-11, TP-13, TP-8, TP-3, and TP-2, and cultivated for 90 days. (**b**,**c**) Ink staining results and resin sections of seedling roots inoculated with TP-8 cultivated for 60 days, revealing abundant formation of pelotons (red arrows), indicating successful fungal colonization. (**d**,**e**) Colonization status of seedlings inoculated with TP-13 and TP-21 after 30 days of symbiosis. (**f**) Illustration of entire root system ink-staining results of seedlings inoculated with Syncom9 after 90 days of symbiosis. The deep-blue areas (green arrows) denote locations of fungal colonization in the roots.

The impact of SAMF on *D. officinale* growth varies significantly (Figure 1a). Specifically, OMFs generally show greater efficacy in promoting seedling growth compared to non-OMFs. Additionally, different mycorrhizal fungi display specific preferences for various seedling growth parameters, with each parameter being predominantly influenced by a particular fungus (Table S4). The *Pletosphaerella niemeijerarum* TP-20, which caused contamination 20 days post-inoculation, was excluded from the analysis. Ink staining confirmed successful colonization by all fungi, with strains TP-2, TP-3, TP-4, TP-7, TP-8, TP-12, TP-13, TP-16, TP-17, TP-18, TP-19, TP-21, TP-22, and TP-24 forming pelotons/peloton-like bodies within 30 days. Strains TP-1, TP-5, TP-6, TP-10, TP-15, and TP-27 exhibited delayed peloton/peloton-like body formation at 90 days (Table S3, Figure 1b–e). Morphological differences in colonization were observed. The control group (CK) lacked pelotons, while Tulasnellaceae and Serendipitaceae fungi formed distinct pelotons (Figure 1b–d). Loose hyphal structures, indicative of initial colonization, as well as hyphal morphology, suggesting senescence, degradation, and complete cell occupation, were observed (Figure 1c,d). However, non-OMFs colonized roots differently, with only a few showing distinct, loosely hyphal structures, while others displayed irregular, tightly coiled hyphal structures (Figure 1e).

### 3.2. The Effect of Mycorrhizal Fungi on the Growth of New Leaves, Roots, and Sprouting Tillers of Seedlings

**Figure 2:**
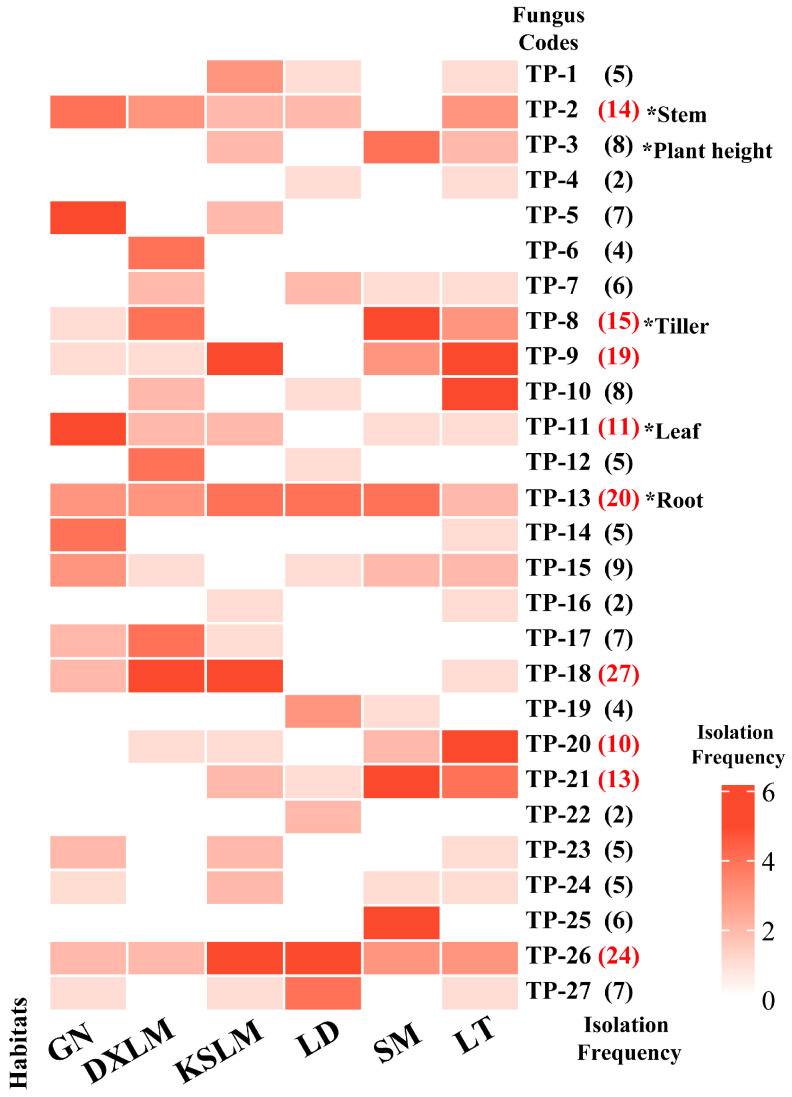
Isolation frequencies of 27 SAMF in six original habitats of *D. officinale*: GN (Guangnan County, Yunnan), DXLM (Langshan Mountain, Hunan), KSLM (Langshan Mountain, Hunan), LD (Luding County, Sichuan), SM (Shimian County, Sichuan), and LT (Luotian Town, Chongqing). Numbers in parentheses indicate the total isolation frequencies of each fungus across the six habitats. Red numerals denote strains with isolation frequencies > 10, reflecting their high prevalence in seedling stage. “*” indicates significant growth promotion, e.g., * leaf indicates significant enhancement of seedling leaf growth; *p* < 0.05.

Seedling growth was significantly correlated with the isolation frequency of mycorrhizal fungi. A significant positive correlation was observed between the growth of new roots and leaves and the isolation frequency of fungi (Figure S1). During the symbiotic cultivation period, the number of new roots, new leaves, and sprouting tillers increased, although the effects varied among fungi. After 30 days, the seedlings inoculated with *Serendipitaceae* sp. TP-14 had the highest number of new leaves (2.626 ± 0.274), while those inoculated with *Trichoderma* sp. TP-23 had the fewest and exhibited a browning of both the medium and roots (Table S4). By 90 days, the number of leaves in the TP-14 inoculation remained stable, whereas seedlings inoculated with *Tulasnella* sp. TP-11 showed a rapid increase in new leaves (4.45 ± 0.462), which was significantly higher than CK (Wald χ^2^= 57.138, *p* < 0.001; Table S4). Notably, TP-11 is commonly found in *D. officinale* native habitats in Guangnan, Luding, and Luotian, with a frequency of isolation occurring 11 times. In contrast, the isolation frequencies of TP-14 and TP-23 were both only five times (Figure 2).

**Figure 3:**
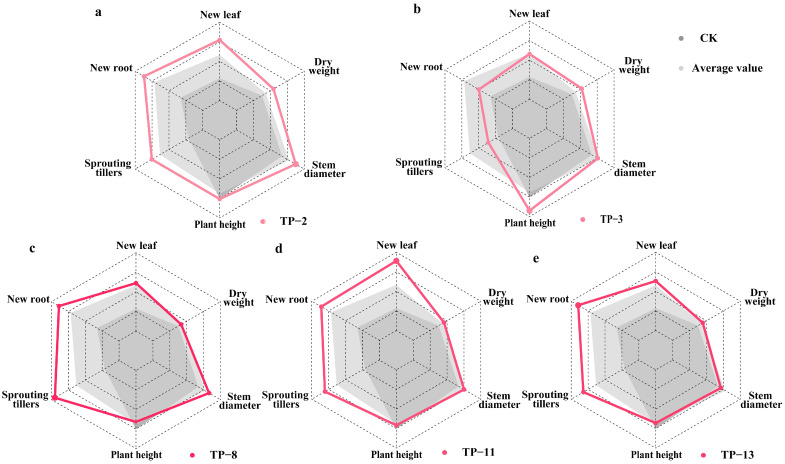
Performance of *D. officinale* seedlings after 90 days of symbiotic cultivation with mycorrhizal fungi TP-2, TP-3, TP-8, TP-11, and TP-13 in terms of various growth parameters. (**a**) TP-2, (**b**) TP-3, (**c**) TP-8, (**d**) TP-11, (**e**) TP-13. Gray-shaded area represents the performance of non-inoculated control group (CK) seedlings. Light-gray-shaded area represents the mean values of growth for seedlings inoculated with all fungal treatments.

The impact on the growth of new roots and sprouting tillers was observed to be gradual. At 30 days, most treatments showed no significant differences. However, by 90 days, the number of new roots in most fungal treatments was significantly higher than CK (*p* < 0.001; Table S4). Seedlings inoculated with TP-14, TP-12, and TP-13 had significantly higher numbers of new roots, reaching 3.084 ± 0.306, 2.738 ± 0.264, and 2.688 ± 0.269, respectively, all significantly higher than CK (*p* < 0.001; Table S4). The isolation frequencies were 5, 5, and 20 times, respectively, for TP-12 and TP-14 sourced from two habitats, whereas TP-13 was found in six native habitats of *D. officinale* (Figure 2). The number of sprouting tillers was consistently the highest in the seedlings treated with *Tulasnella* sp. TP-8, which had an isolation frequency of 15 across four habitats (Figure 2). By the end of the study, TP-8 showed a significant increase in sprouting tillers, reaching 0.949 ± 0.194, which was significantly higher than CK (*p* = 0.018; Table S4). In conclusion, mycorrhizal fungi isolated from multiple habitats, with a high isolation frequency, demonstrated significant growth-promoting functions. Among them, *Tulasnella* sp. TP-11, TP-13, and TP-8 were identified as the core OMFs associated with *D. officinale* seedlings, significantly promoting the growth of new leaves, new roots, and sprouting tillers, respectively (Table S4, Figure 3c–e). These findings highlight the critical role of specific mycorrhizal fungi in the early developmental stages of *D. officinale*.

### 3.3. The Effect of Mycorrhizal Fungi on Plant Height, Stem Diameter Growth, and Dry Weight of Seedlings

Few mycorrhizal fungi enhance stem growth in *D. officinale* seedlings, with relatively slow effects. After 30 days, no significant differences in plant height were observed between treatment groups and CK (*p* > 0.05; Table S4). However, seedlings inoculated with *Tulasnella* sp. TP-13 and *Trichoderma* sp. TP-24 exhibited a significantly greater stem diameter compared to CK (Wald χ^2^ = 70.986, *p* < 0.001), although TP-24 negatively affected root growth later (Figure S3). After 90 days, only seedlings inoculated with *Tulasnella* sp. TP-3 exhibited significantly greater plant height (4.621 ± 0.155 cm) and organic matter accumulation (0.052 ± 0.003 g) compared to other groups (Wald χ^2^ = 192.919, *p* < 0.001; Table S4). TP-3 was isolated eight times from three habitats: Karst landform, Shimian, and Luotian (Figure 2). *Tulasnella* sp. TP-2 significantly enhanced the stem diameter (2.622 ± 0.118 mm) and organic matter accumulation (0.055 ± 0.003 g), which were significantly higher than CK (*p* < 0.001; Table S4). TP-2 was isolated 14 times from Guangnan, Danxia landform, Karst landform, Luding, and Luotian (Figure 2). In summary, *Tulasnella* sp. TP-3 and TP-2 are core OMFs for *D. officinale* seedlings, significantly promoting growth (Figure 3a,b).

### 3.4. The Ability of Synthetic Fungal Combinations to Promote Seedling Growth

**Figure 4:**
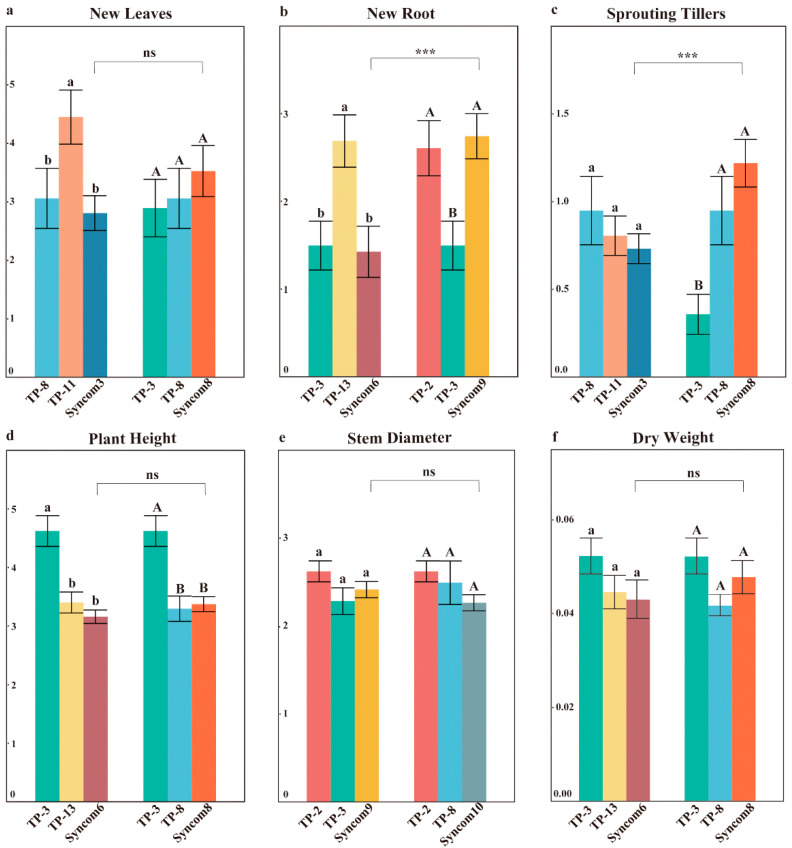
The impact of different synthetic fungal combinations on the growth of *D. officinale* seedlings. (**a**–**f**) Represent the effects of different synthetic fungal combinations on the new leaves, new roots, sprouting tillers, plant height, stem diameter, and dry weight in seedlings of *D. officinale,* respectively. Different uppercase/lowercase letters indicate significant differences among different treatments, *p* < 0.05, “ns” indicates no significant differences among different treatments. “***” indicates significant differences among different synthetic fungal combinations, *p* < 0.001.

Thirty days after inoculation, synthetic fungal combinations successfully colonized the roots of *D. officinale* seedlings. Staining of the entire root system revealed that fungal colonization occurred at multiple, discontinuous, and randomly selected sites (Figure 1f). Overall, synthetic fungal combinations composed of core OMFs did not demonstrate more comprehensive growth-promoting effects compared to single fungi (Figure S2). Syncom8, composed of TP-3 and TP-8, significantly increased the sprouting tillers compared to single fungi but resulted in lower plant height than TP-3 (Figure 4c). There were no significant differences in new leaves between Syncom8 and single fungi (Figure 4a). Syncom6, composed of TP-3 and TP-13, exhibited lower plant height and dry weight compared to single fungi, with significantly fewer new roots than TP-13 (Figure 4b,d,f). Notably, the roles of core OMFs varied among different communities. In the new roots, both Syncom6 and Syncom9 contained TP-3, but Syncom9 with TP-2 produced more new roots (Figure 4b). Similarly, both Syncom3 and Syncom8 contained TP-8, but Syncom8 with TP-3 exhibited higher sprouting tillers (Figure 4c). The functionality of core OMFs changed within synthetic communities.

**Figure 5:**
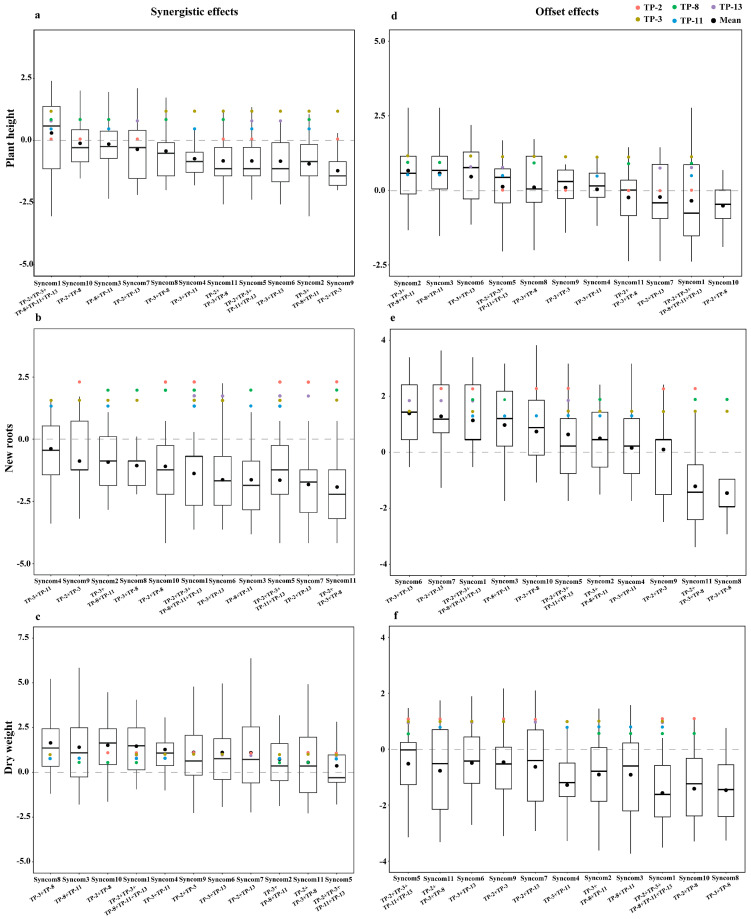
Synergistic and offset effects of synthetic fungal combinations on the plant height, new root, and dry weight in seedlings of *D. officinale*. (**a**–**c**) Synergistic effects, (**d**–**f**) Offset effects. Box plots depict the deviation index between the effects of combination inoculations and the maximum or minimum effects of single fungi inoculations. Colored circles represent the mean effects of single fungi inoculations for each core OMF. Black circles represent the mean effects of combination inoculations.

Evaluating the synergistic and offset effects across various growth parameters among the 11 synthetic fungal combinations revealed that the offset effects predominantly outweigh the synergistic effects within the combinations composed of core OMFs (Figure 5). The offset effects were particularly pronounced in plant height and new root growth (Figure 5d,e). Notably, even Syncom1, which comprises all five core OMFs, demonstrated only a weak synergistic effect on plant height growth (Figure 5a). Synergistic effects on dry weight were exclusively observed in Syncom8, Syncom3, Syncom10, Syncom1, and Syncom4 (Figure 5c).

## 4. Discussion

### 4.1. “Core + Stochastic” Fungal Recruitment

Orchids maintain intricate symbiotic relationships with mycorrhizal fungi throughout their lifecycles, exhibiting remarkable habitat specificity [35]. This ecological pattern likely stems from their “core + stochastic” fungal recruitment strategy, which comprises two complementary processes: (1) phylogenetic conservatism through persistent associations with core fungal lineages (e.g., Tulasnellaceae and Serendipitaceae) providing essential nutritional support and (2) ecological plasticity achieved via random selection of habitat-specific fungi [3,36]. Comparative analysis of fungal communities in *Cyrtochilum retusum* and *Epidendrum macrum* seedlings across different habitats revealed fungal communities consisting of both stable core OTUs and randomly selected habitat-dependent OTUs, providing empirical support for this recruitment strategy [22]. Zhang et al. further elucidated environment-driven fungal association mechanisms through Sanger sequencing, demonstrating significant frequency differences in Serendipitaceae (47.3% vs. 4.8%) and Tulasnellaceae (43.6% vs. 95.2%) between the lithophytic and epiphytic populations of *D. officinale* [17]. Notably, frequently isolated mycorrhizal fungi often exhibit enhanced growth-promoting effects. For instance, the high-frequency fungal isolate DYXY033 (isolation frequency 21.86%) from *Paphiopedilum hirsutissimum* significantly enhanced seed germination and seedling development [37]. Our findings align with these observations, showing habitat-specific fungal community variations in *D. officinale* (Figure 2). Crucially, we identified five *Tulasnella* strains (TP-2, TP-3, TP-8, TP-11, and TP-13) with high-frequency distribution across multiple habitats (>4 habitats) that significantly promoted seedling growth (Figure 3 and Figure S1). These collective results suggest that the “core + stochastic” fungal recruitment strategy in orchids confers functional flexibility in nutrient acquisition, water absorption, and enhanced environmental adaptability/resilience [38,39]. Elucidating the mechanistic roles of core mycorrhizal fungi in orchid growth and ecological adaptation holds critical implications for developing effective conservation strategies.

### 4.2. Functional Diversification of Mycorrhizal Symbiosis

OMFs colonize host roots through root hair penetration or direct epidermal entry into cortical cells, forming tightly coiled hyphal structures termed pelotons [40]. Nutrient transfer primarily occurs during peloton degradation [13,41]. Transcriptional analysis of *Serapias vomeracea* root cells revealed an elevated expression of amino acid transporter genes (*SvAAP1*, *SvAAP2*, and *SvLHT*) in intact peloton cells compared to condensed structures [42]. We observed distinct colonization patterns of OMFs and non-OMFs on the roots of *D. officinale* seedlings. Tulasnellaceae and Sebacinaceae fungi formed pelotons, ranging from loosely organized with clearly defined mycelia to semi-loose and densely packed formations (Figure 1c,d). In contrast, non-OMFs such as *Pletosphaerella cucumerina* TP-21 produced irregular, tightly coiled hyphal structures with multiple staining regions (Figure 1e). The intracellular fungal structures differed morphologically from pelotons and may reflect functional divergence in symbiotic strategies. For instance, while classical OMFs facilitate sustained nutrient transfer (e.g., carbon and nitrogen), non-OMFs like TP-21 may engage in synthesizing phytohormones such as indole -3- acetic acid (IAA) [43], yet ultimately exhibit reduced growth promotion or even detrimental effects on host development (Figure S3). These findings collectively suggest that fungal colonization morphotypes may correlate with functional specialization in nutrient exchange—some specializing in sustained nutrient transfer, others in stress response or pathogen defense, potentially through synergistic fungal networks [44,45].

Previous studies have established that OMFs mediate the transfer of diverse nutrients, particularly carbon, phosphorus, and nitrogen, to their host plants. The functional heterogeneity in fungal nutrient provisioning critically determines host plant growth and ecological adaptation. Stable isotope tracing has revealed specificity in carbon–nitrogen allocation patterns within orchid–fungal symbioses [46,47]. For instance, *Cephalanthera damasonium* acquires 77% ± 3% of nitrogen and 33% ± 10% of carbon from *Cortinarius* and *Thelephora* associates, whereas *Epipactis palustris* obtains merely 30% ± 2% nitrogen while maintaining carbon autotrophy with *Ceratobasidium* and *Leptodontidium* symbionts [46]. Shan et al. demonstrated that *Mycena* sp. MF23 colonization enhances NH_4_^+^/NO_3_^–^ uptake efficiency and upregulates glutamine synthetase/glutamate dehydrogenase activities in *D. officinale* [47]. Our findings extend this functional differentiation to organ-specific developmental regulation. *Tulasnella* strains exhibited specialized morphogenetic effects. TP-2/TP-3 significantly enhanced stem growth, and TP-8 promoted tiller sprouting. Meanwhile, TP-13 and TP-11 preferentially promoted root (2.69 ± 0.26 vs. 0.98 in CK) and leaf development (4.45 ± 0.26 vs. 1.42 in CK), respectively (Figure 3, Table S3). This suggests that fungal-mediated regulation of nutrient allocation coordinates organ-specific morphogenesis. Future studies should prioritize elucidating how these symbiotic partners synergistically enhance orchid environmental adaptation mechanisms.

### 4.3. Synthetic Fungal Combinations: Offset vs. Synergistic Effects

Orchids are associated with diverse fungi in nature, forming complex fungal communities [48,49]. This suggests that fungal communities, while generally more effective than single fungal strains in promoting orchid growth and ecological adaptability, may vary in effectiveness depending on the specific interactions among the fungi within the community [48,49]. Contrary to the prevailing hypothesis that beneficial microbial combinations generate synergistic effects, our study revealed that synthetic fungal combinations constructed from core OMFs consistently underperformed compared to single strains in overall effectiveness (Figure 4, Figure 5 and Figure S2). This performance attenuation likely stems from ecological specialization—fungi from distinct habitats evolve finely tuned interaction networks that resist artificial recombination. Notably, deviations in growth parameters indicated that synthetic fungal combinations exhibited synergistic effects on dry weight, whereas offset effects were more pronounced for new root number and plant height (Figure 5). These findings align with Shao et al., who observed no protocorm development enhancement through symbiotic co-culturing [50], and Hori et al. reported that fungal isolates that enhanced growth in single inoculations did not significantly improve performance in dual inoculations in *Brassica rapa* var. *perviridis* [26]. Intriguingly, parallel patterns emerge in arbuscular mycorrhizal (AM) systems, where single AM fungal inoculants surpass mixed formulations in enhancing both monoterpene production and plant growth [51]. Several mechanistic explanations could account for these results. For instance, the single fungal strains achieve superior root colonization efficiency through co-evolved recognition systems. When multiple growth-promoting fungi compete for overlapping resources (e.g., plant-derived signals or nutrients), offset effects emerge [8], as the TP-3–TP-13 combination exhibited the most pronounced offset effects in new roots, suggesting competition for sharing pathways (Figure 5). Excessive growth-stimulatory signals from multiple highly active fungi may trigger negative feedback regulation in plants. This aligns with observations in *Brassica rapa* var. *perviridis*, where co-inoculation of two strongly growth-promoting fungi reduced the synergistic effects and the increased offset effects, demonstrating no additive effects [26]. The control environments with uniform nutrient supply and stable temperature and humidity failed to replicate the ecological niche segregation that enables fungal complementarity in natural habitats [52,53]. Such conditions suppress habitat-specific fungal adaptations, converting natural collaborators into competitors. Therefore, the efficacy of growth-promoting fungal combinations can be more accurately assessed under specific habitats that preserve ecological interactions.

### 4.4. Perspectives on the Application of Mycorrhizal Fungi

Understanding the mechanisms of orchid–mycorrhizal symbiosis is crucial for both preserving biodiversity through ecological balance maintenance and enabling sustainable agricultural innovation [20]. Our study directly aids biofertilizer development for *D. officinale* by resolving two bottlenecks: (1) identifying stage-specific OMFs, such as Sebacinales LQ for seed germination [11] and *Tulasnella* fungi (core OMFs: TP-2, TP-3, TP-8, TP-11, and TP-13) for seedlings growth, which enables the design of the precision biofertilizers and for practical applications, and (2) providing a theoretical foundation for fungal combination-based biofertilizers through critical considerations in their development. Notably, preliminary investigations further suggest significant differences in the nutrient uptake efficiency of nitrogen (N) and phosphorus (P) among *Tulasnella* strains. For example, under low-N/high-P conditions, TP-13-colonized seedlings developed 9.50 ± 0.29 roots, whereas TP-8 exhibited only 5.15 ± 0.40 roots. Under high-N/high-P conditions, TP-13 root numbers decreased by 30%, while TP-8 increased by 20% (Yang et al., unpublished data). To operationalize these insights, future studies should focus on three key areas. First, analyze fungal nutrient preferences to avoid the offset effects caused by highly growth-promoting strains competing for overlapping resources. Second, prioritizing functional complementarity (e.g., pairing nitrogen-fixing and phosphate-solubilizing strains) over single-strain performance. Third, conducting habitat-specific efficacy evaluations to account for environmental modulation of fungal-plant interactions. OMFs uniquely sustain orchid lifecycles while enhancing medicinal quality (e.g., increasing the polysaccharide content by 42.7% via *T. calospora*) [3], directly addressing industrial cultivation challenges where excessive chemical inputs degrade ecosystems [54,55]. By prioritizing locally adapted strains and fungal–nutrient co-formulations, our framework replaces unsustainable practices with symbiotic systems that synchronize plant–fungal coordination, reducing agrochemical dependence. This paradigm shift toward ecology-driven biofertilizers offers a scalable model for medicinal orchid cultivation.

## 5. Conclusions

This study employed *in vitro* symbiotic experiments to explore the ecological functions of SAMF isolated from diverse habitats. The results demonstrated that mycorrhizal fungi, widely distributed across multiple habitats with high isolation frequencies, significantly promoted *D. officinale* growth, with specific fungi favoring different growth parameters. Specifically, *Tulasnella* sp. TP-2, TP-3, TP-8, TP-11, and TP-13 were found to significantly enhance stem diameter, plant height, sprouting tillers, new leaf, and new root growth, respectively, and were identified as core OMFs for *D. officinale* seedlings. However, contrary to expectations, synthetic fungal combinations composed of these core OMFs exhibited pronounced offset effects rather than synergistic growth-promoting effects. This highlights the complexity of interactions within fungi and emphasizes the need for caution when applying synthetic combinations.

## Data Availability

The original data presented in this study are openly available in the Figshare repository at https://doi.org/10.6084/m9.figshare.27224799 (accessed on 20 February 2025).

## Supplementary Materials

The following supporting information can be downloaded at https://www.mdpi.com/article/10.3390/plants14071024/s1.
