# Supplementary material for: Core Mycorrhizal Fungi Promote Seedling Growth in Dendrobium officinale: An Important Medicinal Orchid
Source: Plants (Basel). 2025 Mar 25;14(7):1024. doi: 10.3390/plants14071024 (PMC11990756; doi:10.3390/plants14071024)
Supplement: Supplementary file 1 [file plants-14-01024-s001.zip › plants-3529838-supplementary.pdf]

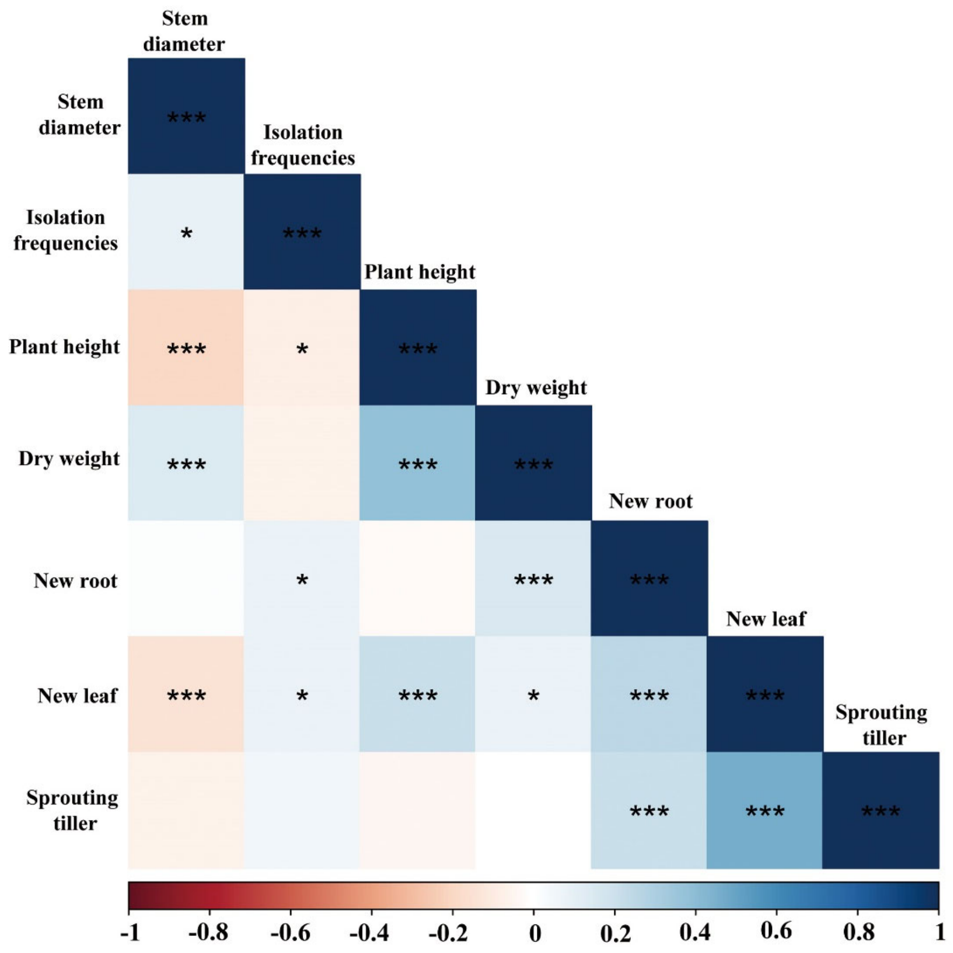

Supplementary Figure S1: Correlation analysis of plant height, stem diameter, number of new leaves, number of new roots, sprouting tiller, dry weight, and fungal isolation frequency across all *D. officinale* seedling samples. "*" *P<0.05*, "***" *P<0.001*

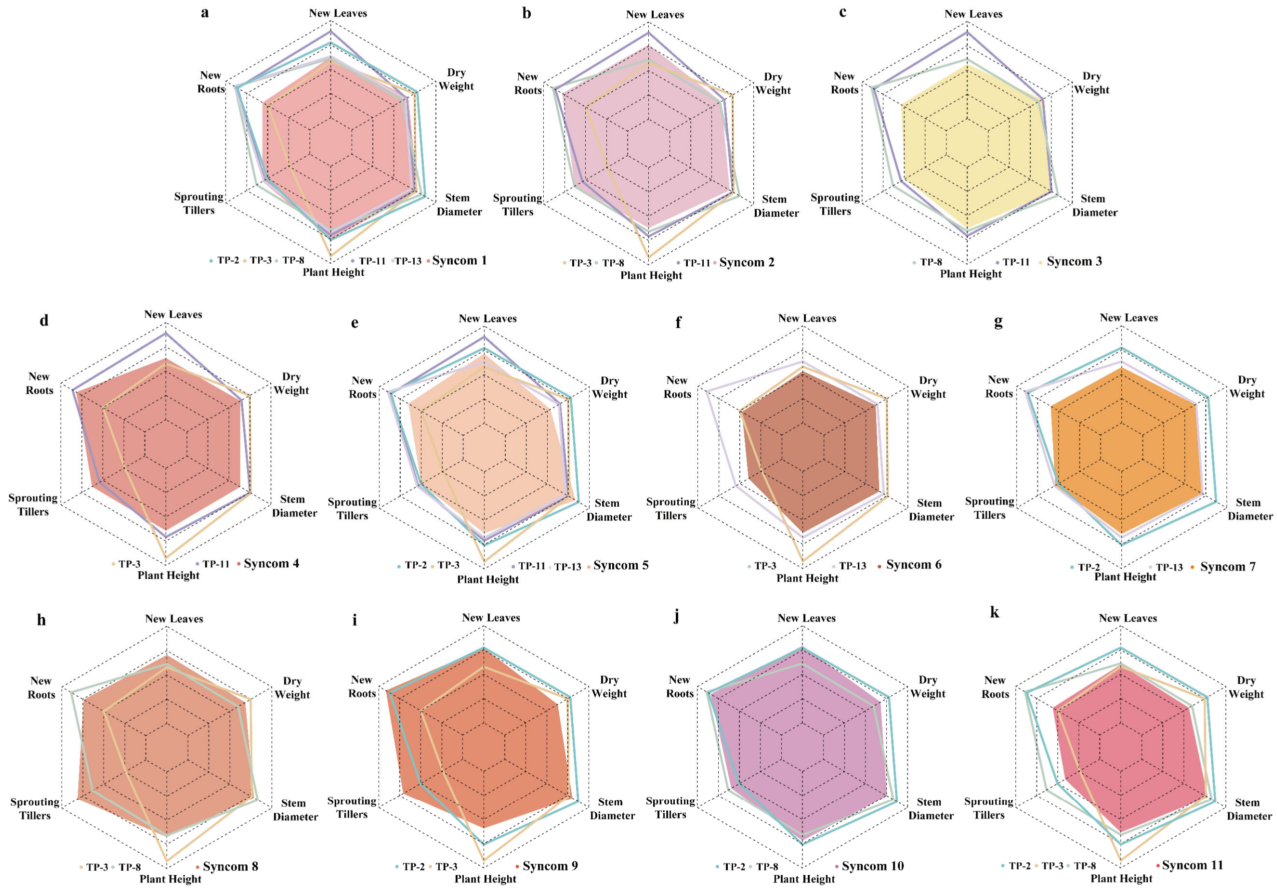

Supplementary Figure S2: Integrated performance of *D. officinale* seedling growth after 120 days of symbiotic cultivation with synthetic fungal combinations. **Note:** Each radar chart includes the effects of a synthetic fungal combinations and single fungi that comprise the combinations on seedling growth. Each colored filled area represents the integrated effect of different synthetic fungal combinations on various growth parameters of seedling. The area formed by each colored line indicates the integrated performance of each single fungi on the various growth parameters of the seedlings.

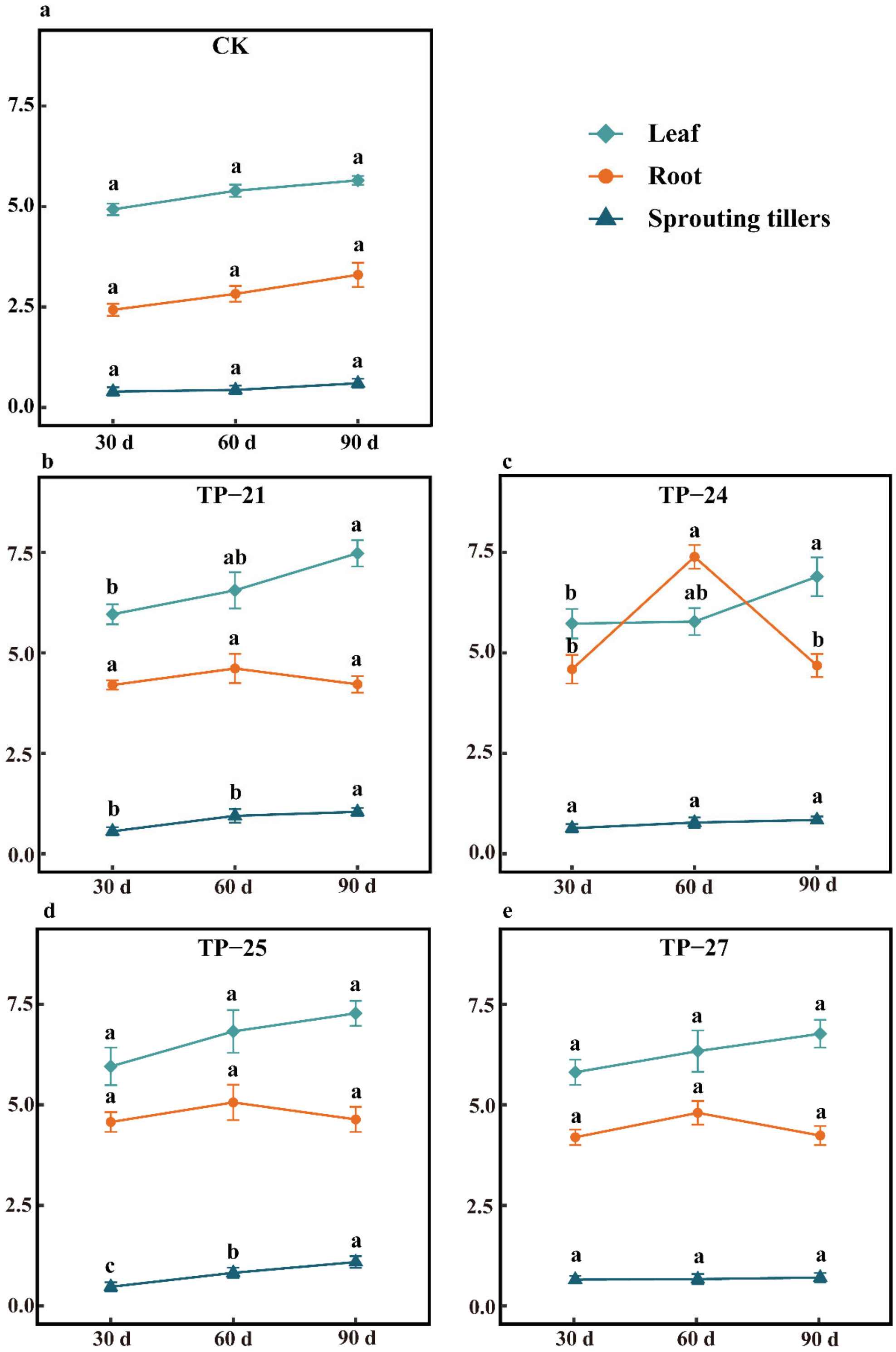

Supplementary Figure S3: Influence of non-OMFs on the leaves, roots, and sprouting tillers of *D. officinale* seedlings.
