# Supplementary material for: Core Mycorrhizal Fungi Promote Seedling Growth in Dendrobium officinale: An Important Medicinal Orchid
Source: Plants (Basel). 2025 Mar 25;14(7):1024. doi: 10.3390/plants14071024 (PMC11990756; doi:10.3390/plants14071024)
Supplement: Supplementary file 1 [file plants-14-01024-s001.zip › Revised Supplementary Table.pdf]

Supplementary Table S1: List of 27 seedling-associated mycorrhizal fungi used to evaluate the effect on *D. officinale* seedling growth, with their isolation frequencies from six original habitats.

**Fungi codes**	**Fungal species**	**GenBank accession number**	**Isolation frequencies from six original habitats**						
			**GN**	**DXLM**	**KSLM**	**LD**	**SM**	**LT**	**Total**
TP-1	*Tulasnella* sp.	MN918475			3	1		1	5
TP-2	*Tulasnella* sp.	MN918476	4	3	2	2		3	14
TP-3	*Tulasnella* sp.	MN918477			2		4	2	8
TP-4	*Tulasnella* sp.	MN918478				1		1	2
TP-5	*Tulasnella* sp.	MN918479	5		2				7
TP-6	*Tulasnella* sp.	MN918480		4					4
TP-7	*Tulasnella* sp.	MN918481		2		2	1	1	6
TP-8	*Tulasnella* sp.	MN918482	1	4			7	3	15
TP-9	*Tulasnella* sp.	MN918483	1	1	6		3	8	19
TP-10	*Tulasnella* sp.	MN918484		2		1		5	8
TP-11	*Tulasnella* sp.	MN918485	5	2	2		1	1	11
TP-12	*Tulasnella* sp.	MN918486		4		1			5
TP-13	*Tulasnella*sp.	MN918487	3	3	4	4	4	2	20
TP-14	Serendipitaceae sp.	MN918488	4					1	5
TP-15	Serendipitaceae sp.	MN918489	3	1		1	2	2	9
TP-16	Serendipitaceae sp.	MN918490			1			1	2
TP-17	*Thanatephorus* sp.	MN918491	2	4	1				7
TP-18	*Fusarium oxysporum*	MN918492	2	7	17			1	27
TP-19	*Clitopilus* sp.	MN918493				3	1		4
TP-20	*Pletosphaerella niemeijerarum*	MN918494		1	1		2	6	10
TP-21	*Pletosphaerella cucumerina*	MN918495			2	1	6	4	13
TP-22	Species of Ascomycota	MN918496				2			2
TP-23	*Trichoderma* sp.	MN918497	2		2			1	5

TP-24	*Trichoderma* sp.	MN918498	1		2		1	1	5
TP-25	*Muscodor* sp.	MN918499					6		6
TP-26	*Muscodor* sp.	MN918500	2	2	7	7	3	3	24
TP-27	*Muscodor* sp.	MN918501	1		1	4		1	7

Original habitats: GN (Guangnan County, Yunnan), DXLM (Langshan Mountain, Hunan), KSLM (Langshan Mountain, Hunan), LD (Luding County, Sichuan), SM (Shimian County, Sichuan), and LT (Luotian Town, Chongqing).

Supplementary Table S2: List of synthetic fungal combinations used to test the effect on *D. officinale* seedling growth

Fungi / SynCom	Habitats	TP-2	TP-3	TP-8	TP-11	TP-13
Syncom1	GN, DXLM, KSLM, LD, SM, LT	+	+	+	+	+
Syncom2	GN, DXLM, KSLM, SM, LT		+	+	+	
Syncom3	GN, DXLM, KSLM, SM, LT			+	+	
Syncom4	GN, DXLM, KSLM, SM, LT		+		+	
Syncom5	GN, DXLM, KSLM, LD, SM, LT	+	+		+	+
Syncom6	GN, DXLM, KSLM, LD, SM, LT		+			+
Syncom7	GN, DXLM, KSLM, LD, SM, LT	+				+
Syncom8	GN, DXLM, KSLM, SM, LT		+	+		
Syncom9	GN, DXLM, KSLM, LD, SM, LT	+	+			
Syncom10	GN, DXLM, KSLM, LD, SM, LT	+		+		
Syncom11	GN, DXLM, KSLM, LD, SM, LT	+	+	+		

Note：“+” indicates that the corresponding fungal strain is included in the synthetic fungal combinations. Habitats refer to the sources of fungal habitats involved in all the fungi within synthetic fungal combinations. GN (Guangnan County, Yunnan), DXLM (Langshan Mountain, Hunan), KSLM (Langshan Mountain, Hunan), LD (Luding County, Sichuan), SM (Shimian County, Sichuan), and LT (Luotian Town, Chongqing).

Supplementary Table S3: The time of finding pelotons/peloton-like bodies in root tissue of *D. officinale* seedlings in fungal treatment group.

**Fungi**	**30 d**	**60 d**	**90 d**	**Fungi**	**30 d**	**60 d**	**90 d**
**TP-1**			+	**TP-14**	+	+	+
**TP-2**	+	+	+	**TP-15**			+
**TP-3**	+	+	+	**TP-16**	+	+	+
**TP-4**	+			**TP-17**	+		+
**TP-5**			+	**TP-18**	+		+
**TP-6**			+	**TP-19**	+	+	+
**TP-7**	+	+	+	**TP-21**	+	+	+
**TP-8**	+	+	+	**TP-22**	+	+	+
**TP-9**		+	+	**TP-23**		+	
**TP-10**			+	**TP-24**	+	+	
**TP-11**		+	+	**TP-25**		+	+
**TP-12**	+	+	+	**TP-26**		+	+
**TP-13**	+	+	+	**TP-27**			+

Note：“+” indicates that the time of finding pelotons/peloton-like bodies. OMFs (TP-1 to TP-16) form “pelotons”, whereas non-OMFs (TP-17-TP-27) form “peloton-like bodies”.

Supplementary Table S4: The effect of mycorrhizal fungi on the growth of *D. officinale* seedlings.

Note：Different lowercase letters indicate significant differences among different fungal treatments, $P<0.05$.

Fungi	New Leaf	New Root	Sprouting Tillers	Plant Height	Stem Diameter	Dry Weight
			**30 Days**			
**CK**	0.475±0.361bc	0.59±0.26ab	0.119±0.134ab	2.683±0.151abcd	1.667±0.13bc	0.015±0.003e
**TP-1**	1.724±0.284abc	1.724±0.205a	0.414±0.105ab	3.014±0.119ab	2.023±0.102abc	0.026±0.002abcd
**TP-2**	1.36±0.275abc	1.196±0.198ab	0.542±0.102ab	2.784±0.115abcd	2.034±0.099abc	0.03±0.002abcd
**TP-3**	2.355±0.3a	1.342±0.216ab	0.435±0.111ab	2.737±0.126abcd	2.099±0.108abc	0.026±0.002abcd
**TP-4**	1.891±0.319abc	1.643±0.23a	0.592±0.118ab	2.463±0.133bcd	1.966±0.115abc	0.039±0.003a
**TP-5**	2.266±0.306ab	1.355±0.221ab	0.417±0.113ab	2.928±0.128abc	2.162±0.11ab	0.032±0.003abcd
**TP-6**	2.336±0.289a	0.999±0.209ab	0.432±0.107ab	3.086±0.121ab	2.056±0.104abc	0.026±0.002abcd
**TP-7**	2.202±0.3ab	1.688±0.216a	0.474±0.111ab	2.902±0.126abcd	2.029±0.108abc	0.038±0.002a
**TP-8**	1.618±0.275abc	1.712±0.198a	0.703±0.102a	2.795±0.115abcd	2.048±0.099abc	0.035±0.002abc
**TP-9**	2.42±0.313a	1.535±0.225ab	0.522±0.116ab	2.733±0.131abcd	1.907±0.112abc	0.031±0.003abcd
**TP-10**	1.426±0.306abc	1.715±0.221a	0.417±0.113ab	2.88±0.128abcd	1.974±0.11abc	0.03±0.003abcd
**TP-11**	2.195±0.319ab	1.252±0.23ab	0.505±0.118ab	2.928±0.133abc	1.934±0.115abc	0.026±0.003abcd
**TP-12**	2.229±0.289ab	1.677±0.209a	0.611±0.107ab	2.818±0.121abcd	1.785±0.104bc	0.026±0.002abcd
**TP-13**	2.31±0.284a	1.621±0.205a	0.552±0.105ab	2.722±0.119abcd	2.375±0.102a	0.022±0.002cde
**TP-14**	2.626±0.306a	1.715±0.221a	0.657±0.113ab	2.802±0.128abcd	1.892±0.11abc	0.031±0.003abcd
**TP-15**	1.495±0.327abc	1.58±0.235ab	0.578±0.121ab	2.909±0.136abcd	2.059±0.117abc	0.034±0.003abcd
**TP-16**	1.146±0.306abc	1.475±0.221ab	0.537±0.113ab	3.281±0.128a	1.578±0.11c	0.029±0.003abcd
**TP-17**	1.6±0.334abc	1.177±0.241ab	0.315±0.124ab	2.171±0.14d	1.999±0.12abc	0.024±0.003bcd
**TP-18**	1.751±0.361abc	0.78±0.26ab	0.14±0.134ab	2.512±0.151bcd	1.973±0.13abc	0.026±0.003abcd

**TP-19**	0.931±0.371abc	1.185±0.268ab	0.166±0.137ab	2.127±0.155d	2.083±0.134abc	0.027±0.003abcd
**TP-21**	1.656±0.306abc	1.091±0.221ab	0.256±0.113ab	2.602±0.128abcd	1.917±0.11abc	0.024±0.003bcd
**TP-22**	1.881±0.295abc	0.928±0.212ab	0.362±0.109ab	2.513±0.123bcd	1.969±0.106abc	0.026±0.002abcd
**TP-23**	0.166±0.371c	0.303±0.268b	0.049±0.137b	2.132±0.155d	1.91±0.134abc	0.03±0.003abcd
**TP-24**	1.423±0.327abc	1.482±0.235ab	0.332±0.121ab	2.735±0.136abcd	1.859±0.117abc	0.032±0.003abcd
**TP-25**	1.648±0.334abc	1.463±0.241ab	0.172±0.124ab	2.286±0.14cd	2.457±0.12a	0.036±0.003abc
**TP-26**	1.996±0.342abc	1.841±0.247a	0.296±0.127ab	2.451±0.143bcd	2.342±0.123ab	0.027±0.003abcd
**TP-27**	1.503±0.3abc	1.084±0.216ab	0.349±0.111ab	2.696±0.126abcd	2.095±0.108abc	0.021±0.002de
			**90 Days**			
**CK**	1.428±0.5c	0.982±0.314gh	0.212±0.151c	3.758±0.181bcd	2.015±0.143c	0.038±0.004bcd
**TP-1**	2.471±0.427abc	2.034±0.269bcdef	0.704±0.129abcd	3.89±0.155abc	2.262±0.122abc	0.041±0.003bcd
**TP-2**	3.872±0.412abc	2.606±0.259ab	0.754±0.125abcd	3.789±0.149bcd	**2.622±0.118a**	0.055±0.003ab
**TP-3**	2.894±0.427abc	1.496±0.269efgh	0.358±0.129cde	**4.621±0.155a**	2.283±0.122abc	0.052±0.003abc
**TP-4**	2.313±0.465abc	2.58±0.292ab	0.806±0.141abcd	3.901±0.169abc	2.194±0.133abc	0.05±0.003abcd
**TP-5**	2.548±0.427abc	2.688±0.269ab	0.397±0.129bcde	3.464±0.155bcde	2.382±0.122abc	0.041±0.003bcd
**TP-6**	3.045±0.445abc	1.909±0.28bcdefg	0.48±0.135abcde	3.972±0.161ab	2.182±0.127abc	0.059±0.003a
**TP-7**	2.891±0.454abc	2.513±0.286abc	0.592±0.138abcde	3.28±0.165bcde	2.514±0.13ab	0.056±0.003ab
**TP-8**	3.06±0.5abc	2.666±0.314ab	**0.949±0.151a**	3.295±0.181bcde	2.493±0.143ab	0.042±0.004abcd
**TP-9**	3.779±0.427abc	2.611±0.269ab	0.666±0.129abcde	4.014±0.155ab	2.157±0.122abc	0.05±0.003abcd
**TP-10**	2.786±0.487abc	2.684±0.306ab	0.547±0.148abcde	3.723±0.177bcd	2.235±0.14abc	0.048±0.004abcd
**TP-11**	**4.45±0.465a**	2.58±0.292ab	0.806±0.141abcd	3.541±0.169bcde	2.257±0.133abc	0.045±0.003abcd
**TP-12**	3.92±0.419ab	2.738±0.264ab	0.86±0.127ab	3.63±0.152bcde	1.936±0.12c	0.039±0.003bcd
**TP-13**	3.163±0.427abc	**2.688±0.269ab**	0.82±0.129abc	3.4±0.155bcde	2.138±0.122bc	0.045±0.003abcd

**TP-14**	2.936±0.487abc	**3.084±0.306a**	0.747±0.148abcd	3.511±0.177bcde	1.973±0.14c	0.043±0.004abcd
**TP-15**	2.015±0.412bc	2.427±0.259abcde	0.682±0.125abcde	3.473±0.149bcde	2.295±0.118abc	0.044±0.003abcd
**TP-16**	4.253±0.476ab	2.511±0.299abc	0.706±0.144abcd	3.962±0.173abc	2.213±0.136abc	0.052±0.003abc
**TP-17**	2.562±0.563abc	2.491±0.354abcd	0.562±0.17abcde	2.963±0.204cde	2.608±0.161a	0.032±0.004d
**TP-18**	2.743±0.476abc	1.939±0.23bcdefg	0.6±0.144abcde	3.248±0.173bcde	2.564±0.136ab	0.037±0.003bcd
**TP-19**	2.508±0.545abc	1.829±0.34bcdefg	0.508±0.165abcde	2.781±0.198e	2.503±0.156ab	0.038±0.004bcd
**TP-21**	3.174±0.454abc	1.109±0.286fgh	0.739±0.138abcd	3.109±0.165bcde	2.25±0.13abc	0.035±0.003cd
**TP-22**	2.559±0.465abc	2.164±0.292abcde	0.877±0.141ab	2.821±0.169e	2.339±0.133abc	0.033±0.003d
**TP-23**	2.133±0.545abc	0.829±0.343h	0.321±0.165de	3.159±0.198bcde	2.526±0.156ab	0.047±0.004abcd
**TP-24**	2.59±0.5abc	1.576±0.31cdefgh	0.538±0.15abcde	2.961±0.181de	2.172±0.143abc	0.039±0.004bcd
**TP-25**	2.968±0.465abc	1.528±0.292defgh	0.787±0.14abcd	2.948±0.169de	2.587±0.133ab	0.039±0.003bcd
**TP-26**	3.177±0.419abc	2.558±0.264ab	0.622±0.127abcde	2.826±0.152e	2.575±0.12ab	0.033±0.003d
**TP-27**	2.46±0.529abc	1.127±0.332fgh	0.402±0.16bcde	3.106±0.192bcde	2.37±0.151abc	0.034±0.004cd
